# On the satisfaction of backbone‐carbonyl lone pairs of electrons in protein structures

**DOI:** 10.1002/pro.2896

**Published:** 2016-02-25

**Authors:** Gail J. Bartlett, Derek N. Woolfson

**Affiliations:** ^1^ School of ChemistryUniversity of Bristol, Cantock's CloseBristolBS8 1TSUnited Kingdom; ^2^ School of Biochemistry, Medical Sciences BuildingUniversity of BristolBristolBS8 1TDUnited Kingdom; ^3^ BrisSynBio, a BBSRC/EPSRC‐Funded Synthetic Biology Research Centre, Life Sciences BuildingBristolBS8 1TQUnited Kingdom

**Keywords:** protein folding, protein structure, protein stability, bioinformatics, hydrogen bonding, noncovalent interactions, n→π* interactions

## Abstract

Protein structures are stabilized by a variety of noncovalent interactions (NCIs), including the hydrophobic effect, hydrogen bonds, electrostatic forces and van der Waals’ interactions. Our knowledge of the contributions of NCIs, and the interplay between them remains incomplete. This has implications for computational modeling of NCIs, and our ability to understand and predict protein structure, stability, and function. One consideration is the satisfaction of the full potential for NCIs made by backbone atoms. Most commonly, backbone‐carbonyl oxygen atoms located within α‐helices and β‐sheets are depicted as making a single hydrogen bond. However, there are two lone pairs of electrons to be satisfied for each of these atoms. To explore this, we used operational geometric definitions to generate an inventory of NCIs for backbone‐carbonyl oxygen atoms from a set of high‐resolution protein structures and associated molecular‐dynamics simulations in water. We included more‐recently appreciated, but weaker NCIs in our analysis, such as *n*→π* interactions, Cα‐H bonds and methyl‐H bonds. The data demonstrate balanced, dynamic systems for all proteins, with most backbone‐carbonyl oxygen atoms being satisfied by two NCIs most of the time. Combinations of NCIs made may correlate with secondary structure type, though in subtly different ways from traditional models of α‐ and β‐structure. In addition, we find examples of under‐ and over‐satisfied carbonyl‐oxygen atoms, and we identify both sequence‐dependent and sequence‐independent secondary‐structural motifs in which these reside. Our analysis provides a more‐detailed understanding of these contributors to protein structure and stability, which will be of use in protein modeling, engineering and design.

## Introduction

Almost 80 years after Pauling and Mirsky predicted the importance of the hydrogen bond in protein structure formation,^1^ the forces governing the folding of a protein's amino‐acid sequence into its three‐dimensional structure are still not fully understood.^2^ Protein structures are stabilized by a variety of noncovalent interactions (NCI) including the hydrophobic effect, van der Waals’ interactions, electrostatic forces, and hydrogen bonds.^3^, ^4^

To complicate matters further, NCIs are context dependent. For example, hydrogen bonds vary in strength depending on the identities and relative geometries of the donor and acceptor groups, and also the local environment.^2^ In addition, weaker donor groups such as Cα‐H and methyl‐H are also possible contributors to protein stability.^5^, ^6^, ^7^, ^8^, ^9^ More specifically, other hydrogen‐bond‐like, NCIs have been implicated, including the *n*→π* interaction^10^, ^11^, ^12^ and methyl‐*π* interactions.^13^, ^14^ These particular interactions are much weaker than canonical hydrogen bonds: the latter are typically worth 3–10 kcal/mol^15^, ^16^; whereas, *n*→π* interactions are estimated at 0.7–1.2 kcal/mol,^10^, ^16^ and methyl‐*π* interactions at 0.9–1.5 kcal/mol.^17^, ^18^ These share common features with hydrogen bonds; notably, the overlap of van der Waals’ radii and orbital overlap, which result in structure stabilization through electron delocalization. Recently, we demonstrated an interplay between hydrogen bonds and *n*→π* interactions,^16^ in particular with asparagine and aspartic acid residues, which form both hydrogen bonds and *n*→π* interactions *via* their side chain carbonyl groups.

Thus, the contributions of and interplay between the various possible NCIs in proteins are complicated, and not straightforward to dissect. However, one thing is clear: for a folded protein to be stable, NCIs must combine to outweigh the contributions to the free energy made up by the entropy lost upon folding and any enthalpically favorable interactions made between the unfolded state and water. In respect of the latter, the degree to which any NCI is made or satisfied relative to the unfolded state is important.

**Figure 1:**
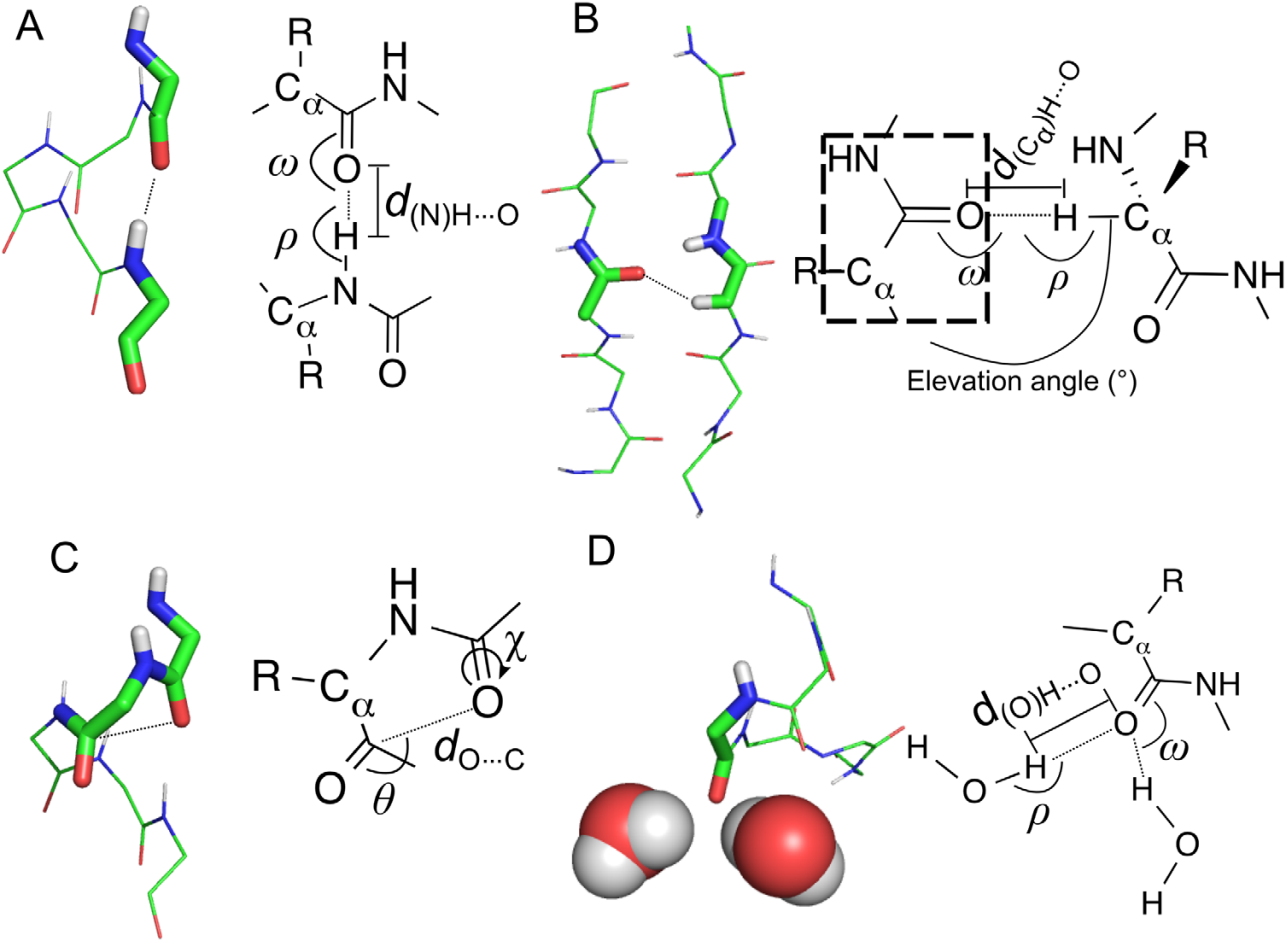
Backbone‐carbonyl‐oxygen non‐covalent interaction (NCI_C=O_) considered in this analysis. (A) “Standard” hydrogen bonds, as exemplified by NH_i_→C=O_i−4_ hydrogen bonds found in an α‐helix (NH_bb_, *d*NH⋯O ≤ 2.44 Å^25^; *ω* ≥ 90°; ρ ≥ 90°). Other donor groups include (*i*) side‐chain NH, e.g., from lysine or arginine, (NH_sc_, parameters as for NH_bb_); and (*ii*), side‐chain hydroxyl groups (OH_sc_, *d*(O)H⋯O ≤ 2.31 Å^25^; *ω* ≥ 90°; ρ ≥ 90°). (B) Hydrogen bonds with a Cα—H group donor (CαH, *d*(Cα)H⋯O ≤ 2.68 Å^25^; *ω* ≥ 90°; *ρ* ≥ 90°, elevation angle < 50°), or alternatively donated by other methyl or ethyl groups from protein sidechains^9^ (CH_X_, parameters as for CαH). (C) n→π* interactions, shown with a main‐chain carbonyl group acceptor (*d*C⋯O ≤ 3.22 Å; 95° ≥ *θ* ≥ 125°; Cα⋯C⋯O⋯H dihedral *χ* ≥ 120°^57^); but these can also have a side‐chain acceptor, e.g., asparagine or glutamine, (n→π*_sc_, parameters as for n→π*). (D) Hydrogen bonds made with water (HOH, *d*(O)H⋯O ≤ 2.31 Å^25^; *ω* ≥ 90°; *ρ* ≥ 90°).

Most commonly, backbone hydrogen bonding in proteins has been depicted quite straightforwardly: NH groups “donate protons” to proximal carbonyl‐oxygen “acceptor” atoms [Fig. 1(A)]; alternatively, this can be viewed as the oxygen atom donating electron density from a lone pair of electrons into the antibonding orbital, σ*, of the N—H bond. Moreover, in each of the two common structures in proteins—the α‐helix and the β‐sheet—each backbone‐carbonyl oxygen atom makes a single such C=O⋯H—N hydrogen bond.^19^ However, these depictions are at odds with the standard model from physical organic chemistry, in which the carbonyl oxygen atom is *sp* ^2^ hybridized, and therefore, presents two lone pairs, either or both of which could participate in hydrogen bonds or other NCIs. Thus, by invoking only one hydrogen bond, and utilizing only one of these lone pairs, the backbone carbonyl atoms of a folded protein could be considered as already unsatisfied as compared with fully solvent‐accessible atoms in the unfolded state. In turn, these lost hydrogen bonds could be considered as adding to the free‐energy debt of the folded state. In support of this, model studies of unfolded alanine peptides reveal an enthalpy deficit for helix formation, which is not provided for by hydrogen bonds,^20^ and which cannot be fully accounted for by modeling interactions of the peptide with water.

The satisfaction of backbone hydrogen‐bonding potential in proteins has been studied.^21^, ^22^ In their *hydrogen‐bonding hypothesis*, Fleming and Rose argue that all potential backbone hydrogen‐bond donors and acceptors are satisfied a significant fraction of the time, either *via* intramolecular hydrogen bonds or hydrogen bonds to water.^23^ The basis of the hypothesis is that unsatisfied hydrogen‐bonding potential is highly unfavorable energetically and therefore rare. Indeed, revisiting foregoing studies, which suggest that up to 10% of this potential remains unmet in folded proteins,^21^ Fleming and Rose show that unsatisfied donors and/or acceptors can be satisfied with small adjustments to the X‐ray crystal structures.^23^, ^24^ However, Fleming and Rose consider carbonyl groups that make just one hydrogen bond to be satisfied, that is traditional hydrogen‐bonded patterns. By extension of their arguments, it stands to reason that if *both* lone pairs could be utilized in hydrogen bonding or other NCIs then the consequences for protein stability would be considerable and favorable.

Herein, we re‐examine the satisfaction of hydrogen‐bonding potential in light of (a) the identification of other and significant NCIs, and (b) the revision of hydrogen‐bonding criteria based on electron‐density topology.^25^, ^26^, ^27^ We explore the question of NCI saturation from the perspective of both lone pairs of electrons of the carbonyl‐oxygen atoms. For example, in an α‐helix, the carbonyl group of residue *i* usually accepts a hydrogen bond from the NH group of the *i* + 4th residue (the traditional depiction), and additionally makes an *n*→π* interaction with the carbonyl group of the *i* + 1th residue,^28^ thereby satisfying both lone pairs. Another means of satisfying both lone pairs in helices comes from bifurcated hydrogen bonds, in which a carbonyl group accepts hydrogen bonds from amides at the i–3th and i–4th positions.^29^, ^30^ For a set of ultra‐high‐resolution protein X‐ray crystal structures, we identify and categorize NCIs made by the carbonyl‐oxygen groups (hereafter referred to as NCI_C=O_). We find that generally, both lone pairs of electrons are satisfied by two NCI_C=O_, and that combinations of different NCI_C=O_ correlate with different secondary structure types. In addition, we use molecular‐dynamics (MD) simulations to explore the dynamics of such NCIs, including examples with under‐ and over‐satisfied carbonyl groups. Although not common, where found the latter are sustained over the course of MD simulations, suggesting that they are pertinent and not structural anomalies. In this way, we identify three structurally conserved NCI_C=O_ motifs that are found in helices. Overall, the system is very much dynamic. Undersatisfied groups are balanced by oversatisfied groups, and the whole system tends towards being slightly oversatisfied.

We believe that this study provides a different and more‐nuanced view of NCIs within protein secondary structures, which is currently not widely considered. It will be of use in the refinement of modeling forcefields for proteins, and to help assess and validate protein models in structure determination, and in protein engineering and design.

## Results

### Data generation

A set of 31 nonredundant, ultra‐high resolution (≤1 Å) structures in which the hydrogen atoms are assigned was obtained from the Protein Data Bank (PDB).^31^ Multi‐chain assemblies were discounted in order to avoid the complication of interchain interactions, which may or may not be due to crystal artefacts. An inventory of NCI_C=O_ made by each residue was generated using operational definitions for four types of NCI (Fig. 1): traditional hydrogen bonds, CH‐based hydrogen bonds, n→π* interactions, and hydrogen bonds made to water.

Not all of the selected protein structures had complete solvent shells. Therefore, each was simulated for 100 ns using a standard molecular dynamics protocol (see Methods for full details). NCI_C=O_ were identified at 1 ns intervals using the same operational definition as for the static structures.

### Backbone carbonyl groups are generally fully satisfied

**Figure 2:**
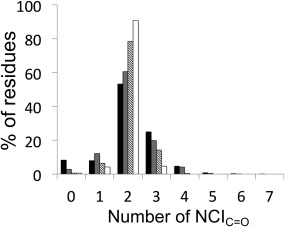
The percentages of NCI_C=O_s per residue made across all residues in proteins. These were measured in three ways: across all residues in the initial, unsolvated high‐resolution crystal structures (black bars); across all residues and snapshots from the last 81ns of a molecular‐dynamics simulation (gray bars); from the distribution of modal averages of all residues across the same set of molecular‐dynamics simulation snapshots (diagonal bars); across all residues and snapshots for those residues that spend at least half of their molecular‐dynamics simulation at their modal average number of NCI_C=O_ (white bars).

Our hypothesis was as follows: given that each carbonyl oxygen atom has two lone pairs of electrons, each of these might be expected to make a NCI. Thus, to be fully satisfied, every backbone‐carbonyl oxygen atom should make two NCIs, one for each lone pair. To begin testing this, we examined the number of NCI_C=O_ made by the carbonyl oxygen atom from the original static protein structures (Fig. 2, black bars). We found that approximately half of carbonyl groups (53%) were satisfied by two NCI_C=O_, and the remainder were under‐ or over‐satisfied.

It is possible that these structures are not all properly solvated, and that a more‐complete picture might be obtained by fully solvating the protein structures ahead of the analysis. In addition, proteins are dynamic systems, and static poses may not reveal the full picture. Therefore, each structure was subjected to MD simulation to enable identification of NCI_C=O_s over a period of time. A disadvantage of using MD forcefields, however, is that necessarily they approximate NCIs. Such parameterization may itself introduce bias into the simulations and how they are interpreted. Hydrogen bonding of carbonyl‐oxygen atoms to water molecules is a case in point: in most forcefields, these are dealt with implicitly rather than explicitly through the application of Coulomb's Law on atomic point charges and the steric bulk of the interacting atoms alone; nonetheless, these tend to result in two hydrogen bonds on average, consistent with each lone pair of the carbonyl oxygen making the hydrogen bonds. Whilst capable of capturing some of the known geometric preferences of hydrogen bonds, these approximations may bias the data away from some of the NCI that might be captured in single high‐resolution structures: however, we could not do the analysis without properly solvated structures, and these could only be reliably obtained by looking at ensembles of MD snapshots. Therefore, we collected data from 81 ns of MD simulation for each structure, taking one snapshot at nanosecond intervals, and then examined the number of NCI_C=O_ made by each carbonyl oxygen at each time‐point in four ways: First, a frequency distribution of the number of NCI_C=O_ made by each carbonyl group in each nanosecond snapshot of the MD simulation showed that 60% of carbonyl groups participated in 2 NCI_C=O_ during the course of their simulation (Fig. 2, gray and Table 1). A smaller, but still significant proportion (∼30%) participated in 1 or 3 NCI_C=O_, and this was close to a normal distribution with a mean NCI_C=O_ of 2, as compared with the static snapshot picture. Second, we looked at the modal average of NCI_C=O_ for each carbonyl group along the length of the simulation (Fig. 2, diagonal lines), which showed that ∼80% of backbone carbonyl groups were fully satisfied, *i.e*., making 2 NCI_C=O_, but with a much smaller contribution from those groups participating in 1 or 3 NCI_C=O_ (6% and 14%, respectively). Finally, when we considered the distribution of numbers of NCI_C=O_ of only those residues that spent half or more of their time through the MD simulations in their modal average state (Fig. 2, white), we found that just over 90% of residues made 2 NCI_C=O_, and that the over‐ and undersatisfied residues balanced out at ∼5% each. For comparison, previous work on forcefield development^32^ has shown that carbonyl‐oxygen atoms in model amides simulated in water have 2 water‐molecule neighbors (equivalent to 2 NCI_C=O_) approximately two‐thirds of the time, with an even distribution between 1 and 3 for the remainder of the time.

**Table 1:** Summary of NCI_C=O_ inventory

(A) Mean number of residues (*n* = 81 MD snapshots) with NCI_C=O_ = *x*. Modal average in parentheses.								
Secondary structure	*x* = 0	1	2	3	4	5	6	Total residues
α‐helix	45 (7)	213 (63)	998 (1390)	584 (580)	163 (17)	17 (0)	0 (0)	2121
β‐strand	31 (9)	226 (163)	732 (965)	204 (86)	23 (0)	1 (0)	0 (0)	1246
3_10_/π‐helix	9 (2)	28 (12)	177 (229)	55 (33)	11 (2)	1 (0)	0 (0)	312
Turn	33 (8)	95 (43)	693 (884)	143 (59)	22 (1)	1 (0)	0 (0)	901
Bend	16 (6)	35 (24)	260 (293)	36 (7)	4 (0)	0 (0)	0 (0)	312
None	22 (3)	65 (41)	435 (507)	60 (8)	5 (0)	0 (0)	0 (0)	550

(B) Total number of each NCI_C=O_ identified (over 81 MD snapshots taken at 1 ns intervals)								
Secondary structure	NH_bb_	NH_sc_	*n*→π*	C_α_—H	O—H	CH_x_	HOH	Total NCI
α‐helix	123568	8148	88839	1493	8315	62514	88088	380605
β‐strand	66133	4026	9772	35391	3366	24373	51239	194300
3_10_/π‐helix	9363	2549	8034	760	968	5378	21267	48319
Turn	17076	9228	18349	3648	2757	116866	94363	162287
Bend	2177	3897	4128	1587	983	5801	36260	58433
None	4724	5642	7115	2964	2447	9482	59711	92085

(C) Average number of each NCI type per residue (mean per snapshot per residue)								
Secondary structure	NH_bb_	NH_sc_	*n*→π*	C_α_—H	O—H	CH_x_	HOH	Total NCI_C=O_ per residue
α‐helix	0.72	0.05	0.52	0.01	0.05	0.36	0.51	2.22
β‐strand	0.66	0.04	0.10	0.35	0.03	0.24	0.51	1.93
3_10_/π‐helix	0.37	0.10	0.32	0.03	0.04	0.21	0.84	1.91
Turn	0.23	0.13	0.25	0.05	0.04	0.23	1.29	2.22
Bend	0.09	0.15	0.16	0.06	0.04	0.23	1.43	2.17
None	0.11	0.13	0.16	0.07	0.05	0.21	1.34	2.07

### Types of NCI made correlate with secondary structure

**Figure 3:**
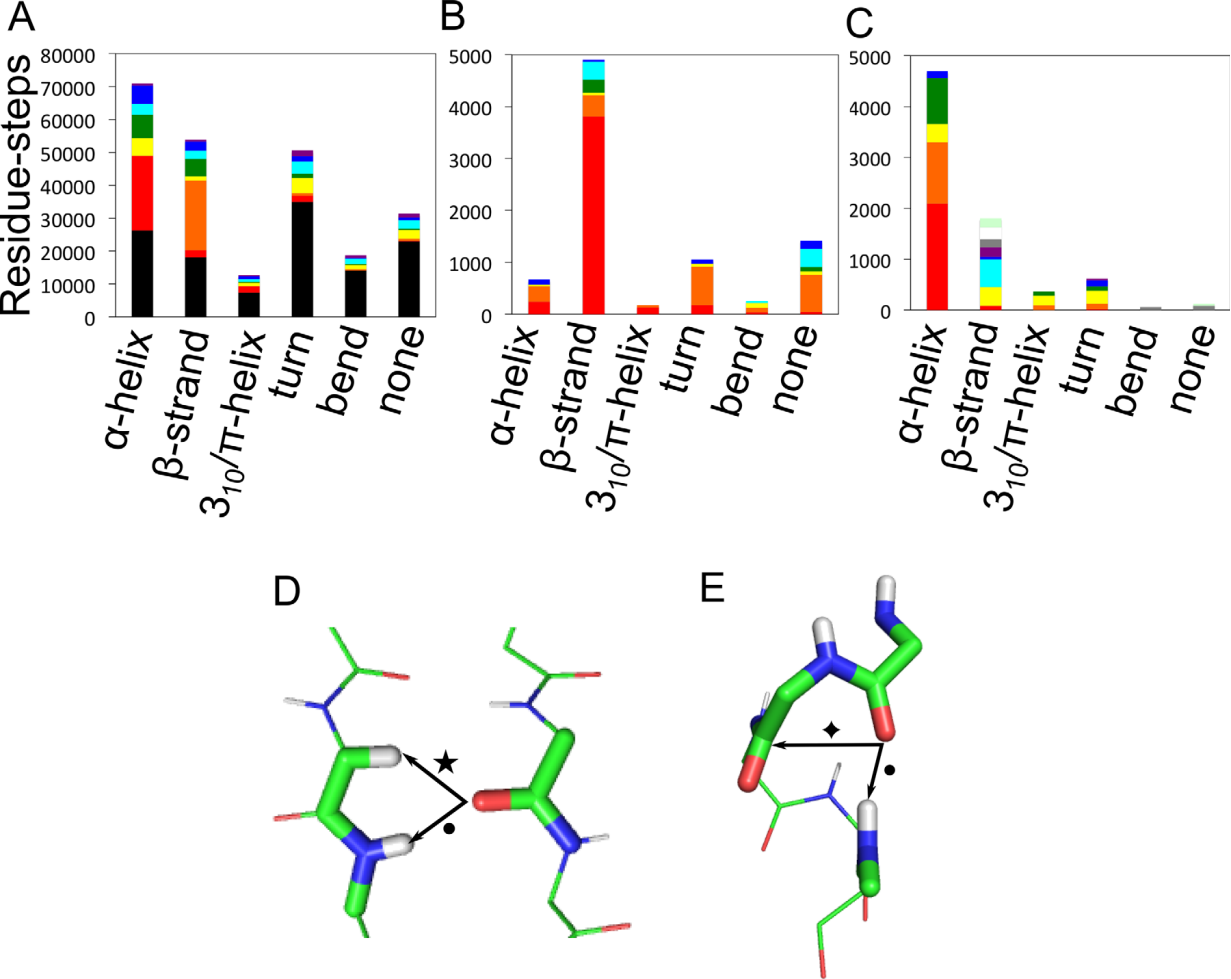
Distributions of types of NCI_C=O_ made in different secondary structure. (A) Where 2 × NCI_C=O_ are made per residue; (B) 1 × NCI_C=O_; and (C) 3 × NCI_C=O_. For clarity, only those combinations of NCI representing at least 2% of all residues are shown in (A), which accounts for 89% of residues overall. Key for panel (A): black bars, 2 × HOH; red, 1 × n→π* plus 1 × NH_bb_; orange, 1 × CαH plus 1 × NH_bb_; yellow, 1 × n→π* plus 1 × HOH; green, 1 × NH_bb_ plus 1 × CH_X_; turquoise, 1 × HOH plus 1 × CH_X_; dark blue, 1 × NH_bb_ plus 1 × HOH; purple, 1 × NH_sc_ plus 1 × HOH. (B and C) Residues were included in the plots for panels (B) and (C) if their modal average number of NCI_C=O_ was 1 or 3, and spent at least 50% of the duration of MD‐simulation in these categories. Key for panel (B): red bars, 1 × NH_bb_; orange, 1 × NH_sc_; yellow, 1 × n→π*; green, 1 × CαH; turquoise, 1 × OH_sc_, blue, 1 × CH_X_. Key for panel (C): red bars, 1 v NH_bb_, 1 × *n*→π*, 1 × CH_X_; orange, 2 × NH_bb_ plus 1 × n→π*; yellow, 1 × NH_bb_, 1 × n→π*, 1 × HOH; green, 1 × *n*→π*, 1 × OH_sc_, 1 × NH_bb_; turquoise, 1 × CαH, 1 × HOH, 1 × NH_bb_; blue, 1 × *n*→π*, 1 × CαH, 1 × NH_bb_; purple, 1 × NH_bb_, 1 × NH_sc_, 1 × *n*→π*; gray, 1 × NH_bb_, 1 × CαH, 1 × CH_X_; white, 2 × NH_bb_, 1 × CH_X_; mint green, 1 × NH_bb_, 1 × NH_sc_, 1 × CαH. (D, E) The most‐common NCI_C=O_ combinations identified in the two most‐prevalent secondary structure types. (D) β‐Strand residues with a backbone NH hydrogen bond (NH_bb_, •) plus a Cα–H hydrogen bond (Cα–H, ★), (PDB 1G66, residues A6, A84‐A85). (E) α‐Helical residues residues with a NH_bb_ (•) plus an *n*→π* interaction (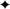), (PDB 1G66, residues A26‐A30). Secondary structures were assigned by Promotif,^42^ which uses a modified version of the Kabsch and Sander DSSP algorithm.^58^ Categories “E” and “B” were combined into a single β‐structure category.

Given the above observation that NCI_C=O_ = 2 for the majority of peptide units, and that this contrasts with traditional models and depictions of regular secondary structures founded on single C=O⋯H—N hydrogen bonds, we asked what types of additional NCIs were being made by the oxygen atoms (Fig. 3).

First, we found that just under half (49%) of all residues in all secondary structure types that made 2 × NCI_C=O_ were fully satisfied by hydrogen bonds to water [Fig. 3(A)]. As might be expected, this proportion was greatest for the nonstructured, bend and turn regions (70%, 72%, and 66% respectively), which are more‐exposed to solvent, and lowest for regular α‐helical and β‐strand conformations (34% and 32%, respectively).

Turning to conformations not wholly satisfied by hydrogen bonds to water, we found that nearly half (44%) of the residues in α‐helical conformations that made 2 × NCI_C=O_ did so with one traditional NH_i_→C=O_i‐4_ hydrogen bond, plus one C=O_i_→C=O_i + 1_ n→π* interaction [Fig. 3(A)]. Approximately equal, but smaller proportions of α‐helical residues, either made one backbone NH hydrogen bond, plus either one hydrogen bond to water (10%), or one CH_X_ (where _*X*_ = 1, 2, or 3) hydrogen bond (14%), or made one n→π* interaction plus one hydrogen bond to water (10%). The preponderance and potential importance of n→π* interactions in the α‐helix has been noted.^13^ However, how these arise is worth reiterating. The NH_i_→C=O_i−4_ hydrogen bonds in α‐helices are unusual: typically, hydrogen‐bond energies are maximized when the angle between the donor and C=O bond axis is ≈120°^33^; however, in the α‐helix this angle approaches ≈180°, *i.e.,* the hydrogen bond is aligned with the C=O bond vector. This results in demixing of carbonyl lone pairs from *sp* ^2^‐like orbitals away from the “rabbit ears” model and into *s*‐type orbital along the C=O bond vector and an orthogonal *p*‐type orbital. The first lone pair participates in the NH_i_→C=O_i‐4_ hydrogen bond, or *n*→σ* interaction, leaving the second lone pair available to make an n→π* interaction with the adjacent carbonyl group.^16^

Our analysis also revealed that half of carbonyl groups found in β‐structure not satisfied by hydrogen bonds to water were satisfied by one backbone NH hydrogen bond (NH_bb_), plus one C_α_‐H hydrogen bond (CαH), (55%), Figure 3(A). Both of these bridge strands [Fig. 3(D)], in what are termed *i → j* interactions. Although the role of CαH interactions has been identified in several studies,^5^, ^34^, ^35^ the consensus is that they are weak, and of lower importance than hydrogen bonds with traditional donors, i.e., protons attached to electronegative nitrogen and oxygen atoms. However, our data, which show that CαH interactions are made by most residues in β‐sheets, suggests that they are common and made significant proportion of the time. Thus, they could also be important contributors to protein stability. Moreover, they help account for the full satisfaction of the carbonyl‐oxygen lone pairs of electrons.

### Under‐satisfied residues participating in 1 NCI_C=O_

As argued by Rose and colleagues,^36^ backbone polar groups that are under‐satisfied in their hydrogen‐bonding potential almost certainly disfavor protein folding by reducing protein stability. Our hypothesis and consideration of both lone pairs on carbonyl oxygen atoms potentially increases the number of such unsatisfied groups. We investigated these by considering residues with a modal average number of just 1 NCI_C=O_, with the additional requirement that the residue had to maintain this number in at least half the snapshots taken from the MD simulations. This was done to ensure that we were considering sustained interactions, and not ephemeral arrangements that may have arisen as the simulations fluctuated.

Figure 3(B) shows that the largest contribution of residues of this type, approximately half, are in β‐structure and make a single NH_bb_. Indeed, across all secondary structure types, a single hydrogen bond made to an NH group (red and orange bars), either backbone or side chain, accounts for 79% of all residues in this NCI_C=O_ = 1 category.

It is interesting to speculate whether these residues do make other, as yet unforeseen NCI_C=O_. We found that the C=O groups in this category regularly made sub‐van der Waals’ contacts with the backbone amide proton of the same residue, and/or with the C_α_ proton of the adjacent residue (Fig. S2, Supporting Information). Neither of these potential NCIs were formally considered in our analysis as they have not been previously documented or recognized as stabilizing; although, weakly stabilizing NCIs between C=O and NH groups have been observed in small‐molecule systems.^37^, ^38^

Additionally, in the nonstructured regions and in turns, we see a larger preponderance of hydrogen bonds donated by a side‐chain NH group. This highlights the importance of side chain—main chain interactions in these regions and has been noted by others (e.g., Refs. 39, 40, 41).

### Over‐satisfied residues participating in 3 NCI_C=O_

**Figure 4:**
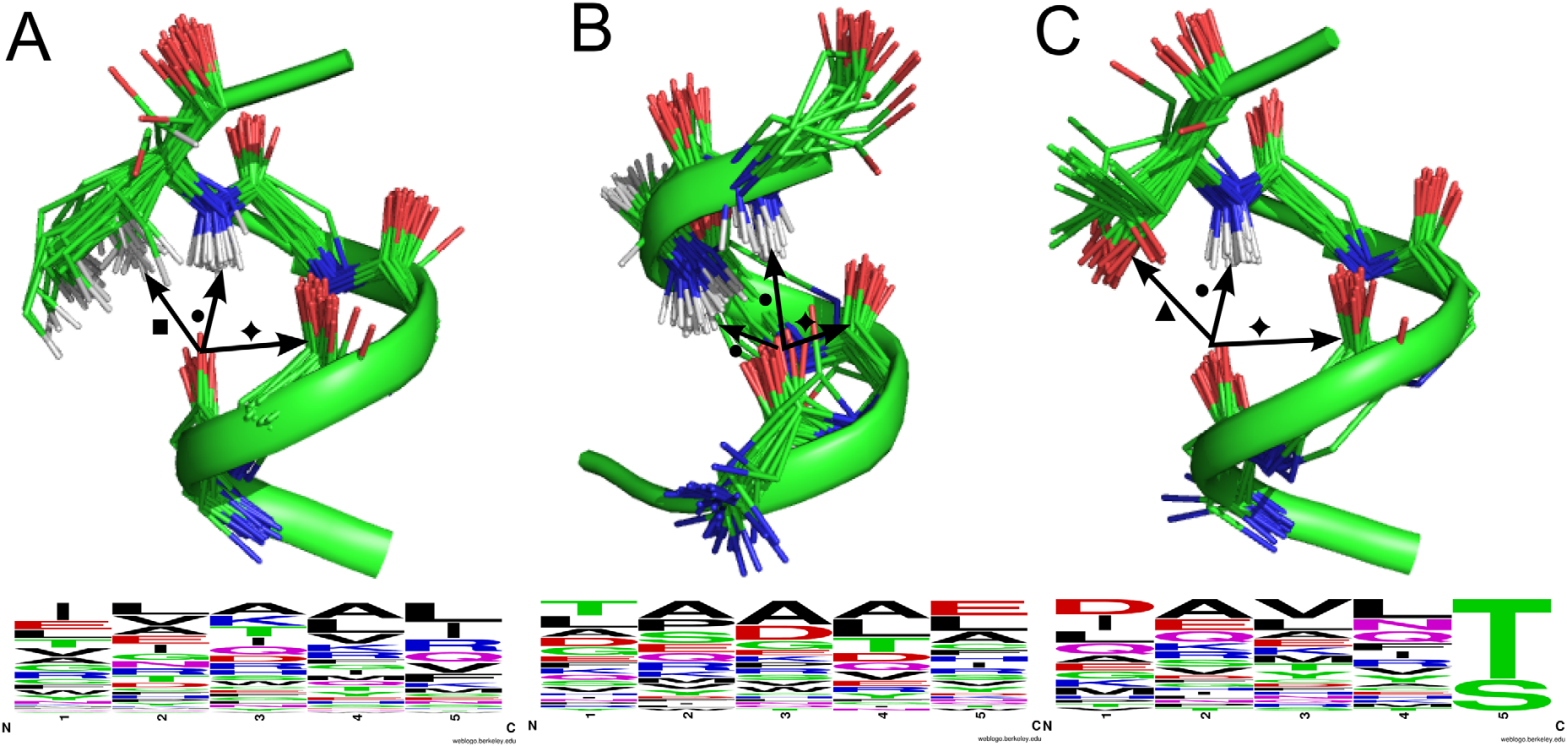
Local structures with over‐satisfied backbone‐carbonyl‐oxygen atoms, i.e., with 3 × NCI_C=O_. (A) α‐helical motifs with 1 × NH_bb_ (•), 1 × *n*→π* interaction (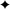) and one 1 × CH_X_ (▪). The residue providing the CHx has been truncated for clarity. (B) Motifs at the α‐helical Ntermini with 2 × NHbb (•) plus 1 × *n*→π* interaction (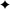). (C) α‐helical C‐termini with 1 × OH_sc_, (▲), 1 × NH_bb_ (•) and 1 × *n*→π* interaction (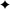), and associated WebLogos^59^ indicating the amino‐acid frequencies from sequences in our dataset that display this motif. Structural images prepared with PyMOL (http://www.pymol.org). PDB codes and residue identifiers for each example can be found in the Supporting Information.

Under‐satisfied NCI_C=O_s are one thing, but residues that make more than two NCI_C=O_ are curious given that there are just two lone pairs of electrons per carbonyl group. Intrigued by the significant proportion (14%) of these over‐satisfied C=O groups, we investigated them by considering only residues where the modal average NCI_C=O_ was 3 in the MD simulation, and again, stipulating that the residue had to be in this state for at least 50% of the snapshots taken from the simulations [Fig. 3(C)]. This identified 11,843 snapshots from 263 individual residues. Three significant groups emerged, all involving α‐helical residues. The largest group formed one NH_bb_ plus one n→π* interaction and one CH_X_ hydrogen bond [63 unique examples from 2391 snapshots, Fig. 4(A)]. These were found at all positions across α‐helices and showed no preference for termini.

The second largest group of over‐satisfied residues formed two NH_bb_, plus an additional n→π* interaction [42 unique examples from 1435 snapshots, Fig. 4(B)]. These were found in α‐helical structures, with two‐thirds coming from the “little h” category defined by Promotif,^42^ i.e., the first or last turn of an α‐helix. The majority were found at the *N*‐termini of α‐helices, where they may have a sequence‐independent role in helix‐capping [Fig. 4(B)]; that is, different from other identified capping motifs, which involve side chain—main chain contacts. The overwhelming majority of these formed bifurcated hydrogen bonds, with donors coming from the i–3rd and i–4th residue. Over all residue‐steps, these accounted for 19.4% of all hydrogen bonds to main‐chain amide groups. Interestingly, when these interactions did fluctuate down to two NCI_C=O_ in the MD simulations, it was usually one of the NH_bb_ that was lost, and not the n→π* interaction, which perhaps runs contrary to expectations given that the latter is considered the weaker of the two interactions.^16^

A third type of three‐NCI_C=O_ cluster was found in the *C*‐terminal turns of α‐helices [31 unique examples from 1039 snapshots, Fig. 4(C)]. This comprised one NH_bb_, a hydrogen bond donated by a side‐chain hydroxyl group (OH_sc_), and an n→π* interaction. Both the OH_sc_ and the NH_bb_ were donated either by serine or threonine residues. This helix‐capping motif has been identified by Richardson & Richardson, who note both hydrogen bonds, but not the additional n→π* interaction.^43^

A small subset of residues (13 unique examples from 567 snapshots) that form 3 × NCI_C=O_ was found in the general β‐strand/extended secondary structure class. These have one NH_bb_, one CαH and an additional hydrogen bond to water. Although these are not well conserved structurally, owing to the different underlying structures found in parallel and antiparallel β‐sheets, similarities can be identified within these groups: they occur in exposed β‐strands where a backbone carbonyl group is exposed and makes a close contact with a water molecule in addition to the NH_bb_ and CαH interactions.

### Prevalence of “weaker” interactions

It is interesting to note the prevalence of weaker interactions found in this study and how they compare with other foregoing studies. We found *n*→π* interactions in 31% of residue‐steps, which agrees with the average of 34% found previously.^12^ Ten percentage of the Cα‐H groups made hydrogen bonds to C=O groups, which is in line with the proportion identified by Derewenda *et al*.^5^ Turning to CH_X_ bonds (donated by side‐chain CH_3_, CH_2_, or CH groups), we find that 10% of all such available groups in the dataset formed these weak hydrogen bonds, a much reduced proportion compared with the 36% found by Yesselman *et al*.^9^ However, this discrepancy can probably be explained in that our analysis only considers hydrogen bonds accepted by main‐chain C=O groups and not other hydrogen bond acceptors.

## Discussion

The analysis that we present provides an inventory of non‐covalent interactions (NCIs) for backbone‐carbonyl oxygen atoms in high‐resolution protein structures. Previous analyses have been dismissed as “apples and oranges” comparisons of hydrogen bonds due to the range of strengths that these can have depending on their environment.^36^, ^44^, ^45^ However, as we consider the satisfaction of lone pairs of electrons via several possible NCIs, rather than simply counting “traditional” hydrogen bonds, we suggest that our analysis offers a different perspective on understanding the stabilization of protein structure, and that this helps to explain certain anomalies of previous models. Key points of our hypothesis are that backbone‐carbonyl oxygen atoms can make up to two NCIs, by virtue of their two available lone pairs; and that ideally both of these should be satisfied in the folded state, as presumably they are both involved with hydrogen bonding to solvent in the unfolded state. Thus, if left unsatisfied the stability of the folded state will be sub‐optimal. This is the case in the more‐common models of regular protein secondary structures, which depict just one C=O⋯H—N hydrogen bond per residue.

In support of our hypothesis, we find that the majority of backbone‐carbonyl oxygen atoms do indeed form two NCIs. This is true for static X‐ray crystal structures of proteins. Moreover, these interactions persist during MD simulations. We categorize the various types of additional NCIs as fully as possible, and in the context of known NCIs over and above C=O⋯H—N hydrogen bonds. Table 1 provides a summary of NCIs identified by secondary structure type; a full breakdown per structure is given in the Supporting Information. We find correlations between local backbone structure and the type of NCI made, which we propose further stabilize the secondary and tertiary structures. These observations were largely independent of side‐chain. Specifically, in addition to two bifurcated hydrogen bonds, carbonyl groups in α‐helices tend to make an n→π* interaction; whereas, in β‐structure (parallel or antiparallel) the second lone pair of electrons of the carbonyl group is satisfied through C=O⋯H—C hydrogen bonds.

In addition, we identify and examine examples of residues that appear to be over‐satisfied; that is, where the number of NCI_C=O_ is greater than two. These account for 14% of all residues in our dataset (judged by modal average). These clustered interactions tend to persist during the lifetime of MD simulations, which suggests that they are not structural anomalies. Interestingly, the most‐prevalent clusters are found in helices, and the most‐frequent of those found at helical termini appears to be sequence‐independent, unlike most helix‐capping motifs previously identified.^43^, ^46^ A smaller proportion of residues (6%) appear to be under‐satisfied in terms of NCI‐making potential. Proteins systems are clearly dynamic, and therefore we expect a distribution of NCI_C=O_ across all residues, and ideally, it should be balanced. Our analysis points to slight oversatisfaction: it is possible that this is due to errors in the way we have assigned NCI_C=O_, or that we are not counting other, as yet unidentified, stabilizing interactions. Interestingly, removing some of the fluctuations from the system—by considering only those residues that spend at least half the simulation time with their modal average number of NCI_C=O_—the systems balance with a 5:90:5 ratio of 1, 2 and 3 NCI_C=O_ made, respectively.

Overall, we can define a *density of NCIs* made by backbone carbonyl groups (*NCI_C_*_=__*O*_, Table 1). On average across all of the proteins that we analyzed this is 2.12 per residue, which rises to 2.22 per residue for the α‐helical regions, and falls to 1.93 per residue in parallel and antiparallel β‐sheets. For comparison, the average numbers of hydrogen bonds made per residue in our data set (excluding weak C—H hydrogen bonds) are 1.42, 1.33, and 1.24 for these three structural classifications, which is greater than that identified by McDonald and Thornton (mean 1.16 H bonds per backbone C=O).^21^ Most likely, this discrepancy arises from the use of updated hydrogen‐bonding criteria, and potentially more‐accurate hydrogen‐atom placement in crystal structures and simulations. Such metrics will hopefully help the quest of seeking a quantitative dissection and description of protein stability.

Traditional textbook and literature descriptions of protein folding that cite hydrogen bonds as one of the major stabilizing determinants of protein secondary structures.^2^, ^4^, ^22^ Our analysis is not at odds with this view, but we believe the picture is more detailed and subtle than often portrayed. Recently, others have demonstrated that, for the α‐helix in particular, the classical model of NCIs may not always be appropriate. Kuster *et al*.^29^ have demonstrated that a slight crankshaft rotation of backbone torsion angles in protein helices accommodates bifurcated hydrogen bonds, in which one backbone amide makes a hydrogen bond to two carbonyl groups, at the *i* + 3rd and *i* + 4th carbonyl groups, without moving the C_α_ and C_β_ atoms from their positions in a classical Pauling α‐helix. These bifurcated hydrogen bonds contribute to the satisfactions of both lone pairs in helices; however, they do not consider other weak NCIs such as the n→π* interaction. Interestingly, they find that 18.5% of helical hydrogen bonds are bifurcated: our data concur with this, but we find that additionally the majority of carbonyl groups making bifurcated hydrogen bonds make an additional n→π* interaction.

Specifically, weaker NCIs such as n→π* interactions and Cα—H hydrogen bonds need to be included to satisfy fully the lone pairs of electrons associated with backbone‐carbonyl oxygen atoms; and the dynamics of the biomolecular systems must be considered. Given the preponderance of these weak interactions, and that they may well be even more readily formed and broken than traditional hydrogen bonds, their roles in protein structure, dynamics, and function may be far reaching. That said, and for the same reasons, gaining thorough experimental, computational and quantitative grasps of these other NCIs will be challenging. Of course, there are concerns and potential caveats in our view and analyses that will need refinement. For example, it is not immediately clear how a model based on satisfying two lone pairs of electrons accommodates carbonyl groups that make 3 NCIs, although there is a dipolar resonance structure for the amide group that places three lone pairs on the carbonyl oxygen. This raises the question of how best we should define and measure NCIs, and, of course, how do we model and assess them computationally and quantitatively. For example, recent work on the Rosetta forcefield has demonstrated that simultaneously modeling the electrostatic and covalent properties of hydrogen bonds improves protein‐structure prediction,^47^ and work on polarizeable, multipolar forcefields such as AMOEBA^48^ has challenged the notion of linear hydrogen bonding in α‐helices. Further quantification of the contributions from each NCI and how they cooperate should inform the development of more‐accurate forcefields for molecular modeling and mechanics, and thus afford a deeper understanding of protein structure and stability.

## Methods

### Inventory generation

The inventory of NCI_C=O_ made by each residue was generated using a python script that measured interatomic distances, angles, and dihedrals, and assigned NCI_C=O_ based on the operational definitions of NCI shown in Figure 1.

### MD simulation

To give each protein structure a full solvent shell, each was simulated in a box at least 2 nm larger than the protein in each direction, filled with TIP3P water,^32^ using the amber99sb‐ildn forcefield^49^ as implemented in the Gromacs‐4.5.3 suite of MD software.^50^ Random water molecules were replaced by sodium and chloride ions to give an overall neutrally charged system with an ionic strength of 0.15*M*. Each simulation was subjected to 2000 steps of energy minimization using steepest descents prior to the MD simulation.

Simulations were performed at 293 K using periodic boundary conditions. Short range electrostatic and van der Waals’ interactions were truncated at 1.4 nm, while long‐range electrostatics were treated with the particle‐mesh Ewald's method,^51^ and a long‐range dispersion correction was applied. Pressure was controlled by Berendsen's thermostat^52^ and temperature by the V‐rescale thermostat.^53^ Simulations were integrated with a leap‐frog algorithm over a 2 fs timestep, constraining bond vibrations with the P‐LINCS method^54^ and water bonds and angles using the SETTLE method.^55^ An initial 200 ps simulation was performed in each case with the protein heavy atoms restrained to their initial co‐ordinate positions to relax the system, before a 100 ns period of unrestrained MD. RMSD profiles of MD trajectories were manually inspected for any significant drift from the original structure (Fig. S1, Supporting Information). PDB snapshots were taken from the trajectory at 1 ns intervals from 20–100 ns, to avoid any bias from initial equilibration.

NCI_C=O_ at each time‐point were identified with the same python script used to interrogate the static structures. Results were stored in a relational database for ease of repeated queries (File 1, Supporting Information). The assumption was made that all carbonyl oxygen atoms interacting only with water (i.e., those that were completely exposed) made two hydrogen bonds with water. This avoided any bias resulting from the water model used, as it has been shown recently^56^ that proteins are under‐solvated in MD simulations.

## Supporting information

Supporting Information
