# Supplementary material for: On the satisfaction of backbone‐carbonyl lone pairs of electrons in protein structures
Source: Protein Sci. 2016 Feb 25;25(4):887–97. doi: 10.1002/pro.2896 (PMC4941217; doi:10.1002/pro.2896)
Supplement: Supplementary file 1 — Supporting Information [file PRO-25-887-s001.docx]

**On the satisfaction of backbone-carbonyl lone pairs of electrons in protein structures**

**Bartlett & Woolfson**

**SUPPLEMENTARY MATERIAL**

**Supplementary Figure 1.**

RMSD plots of each molecular dynamics trajectory. RMSD was calculated over all protein atoms from a least-squares-fit to backbone atoms of the reference structure.

**
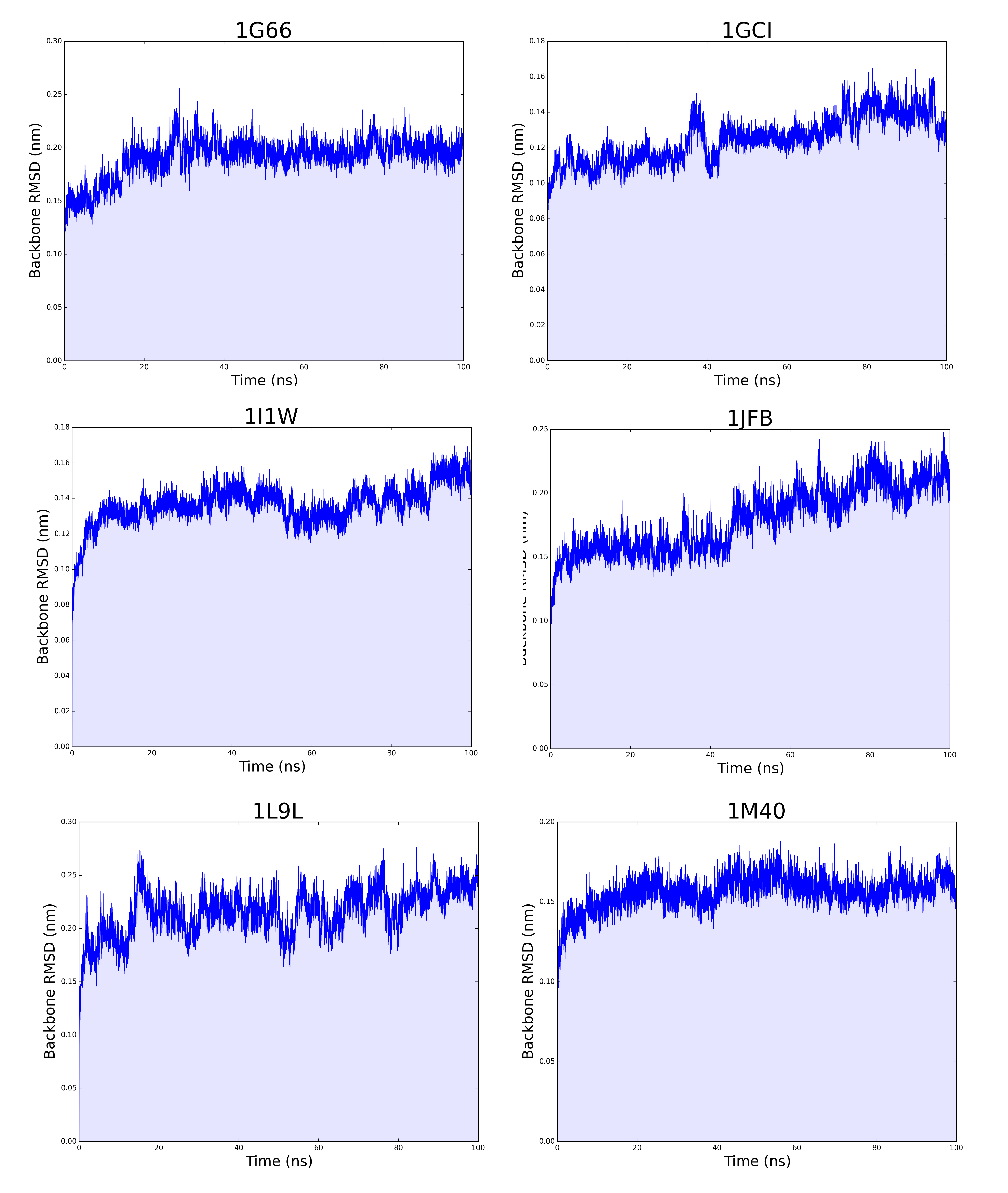
**

**
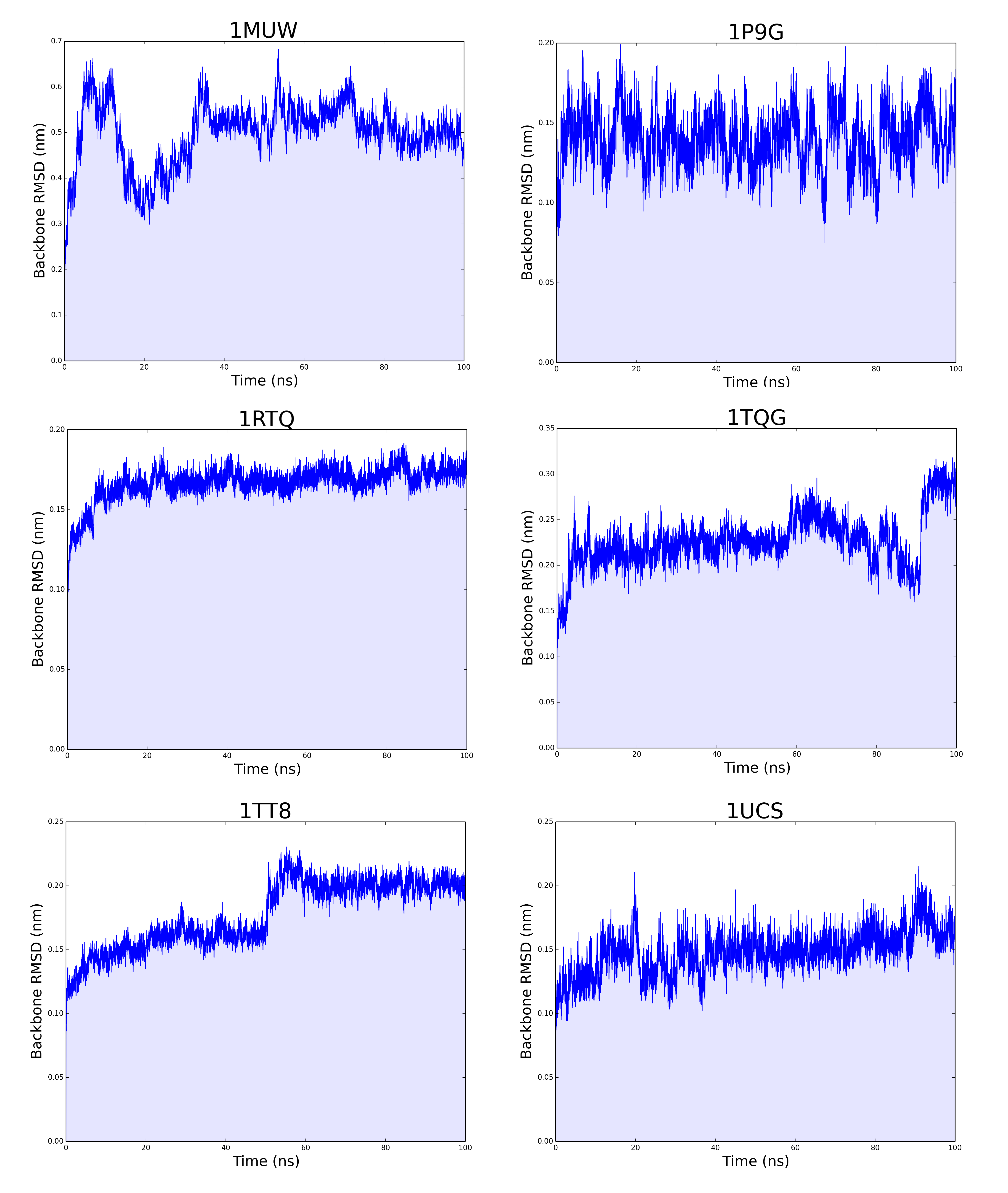

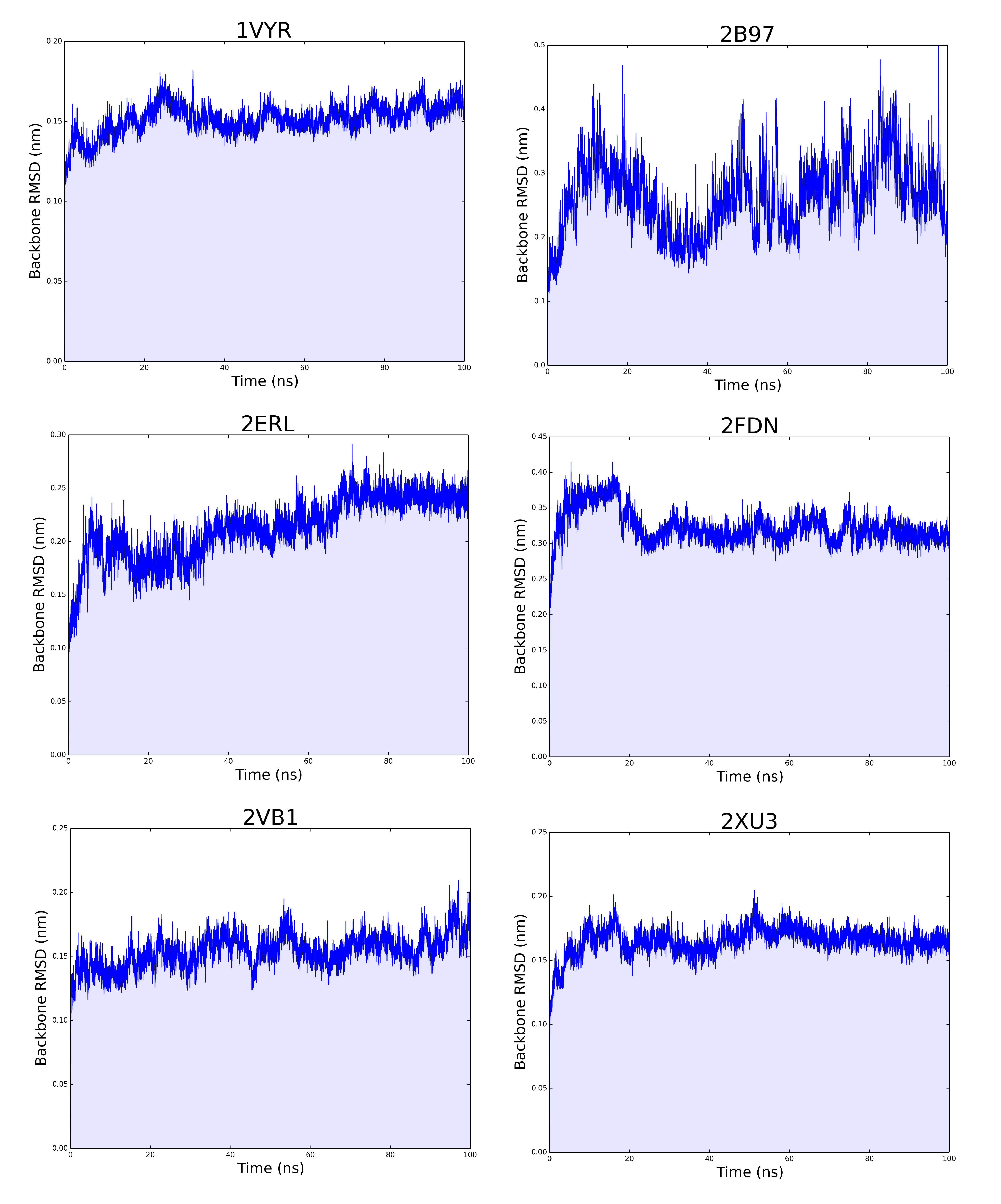

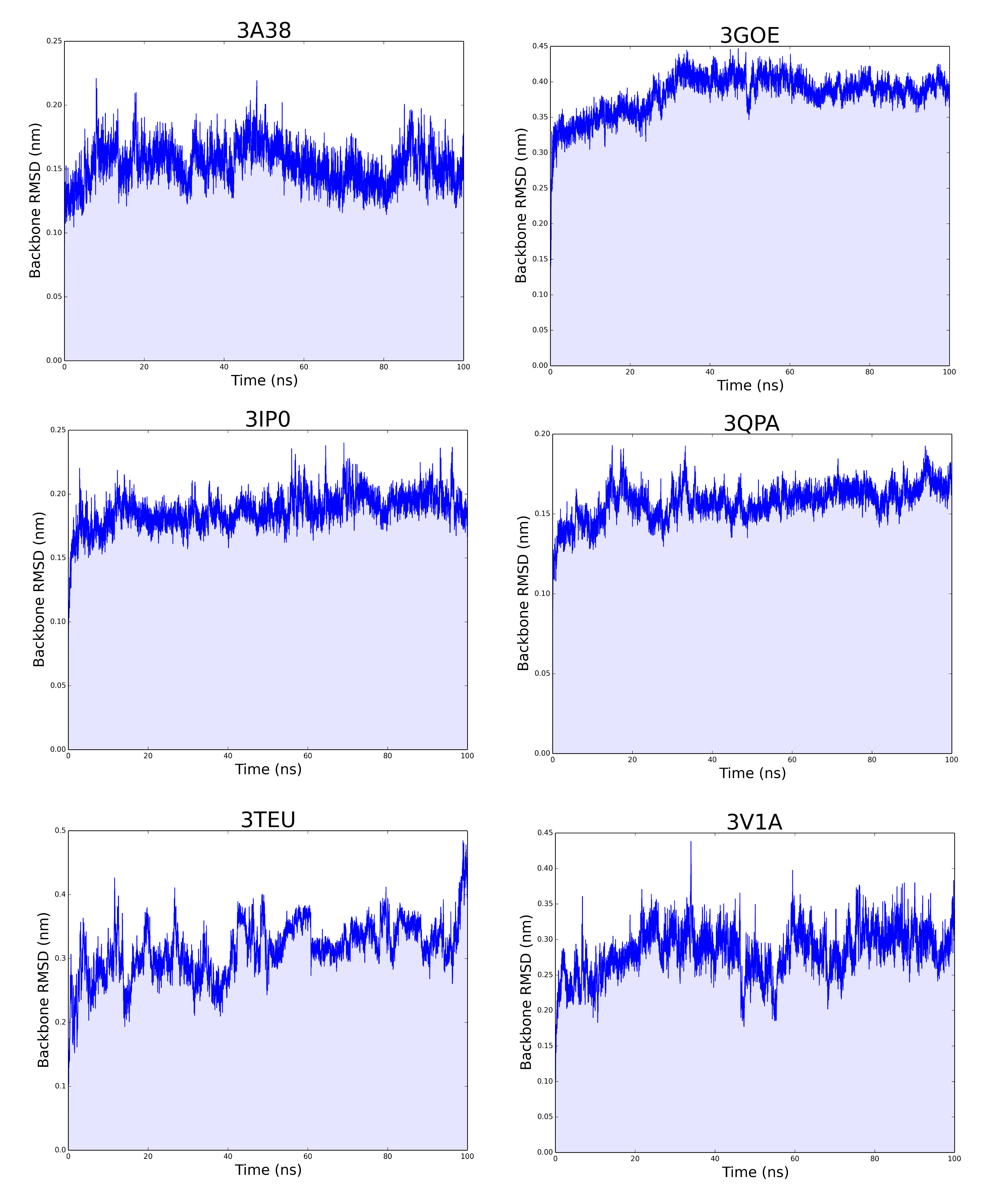

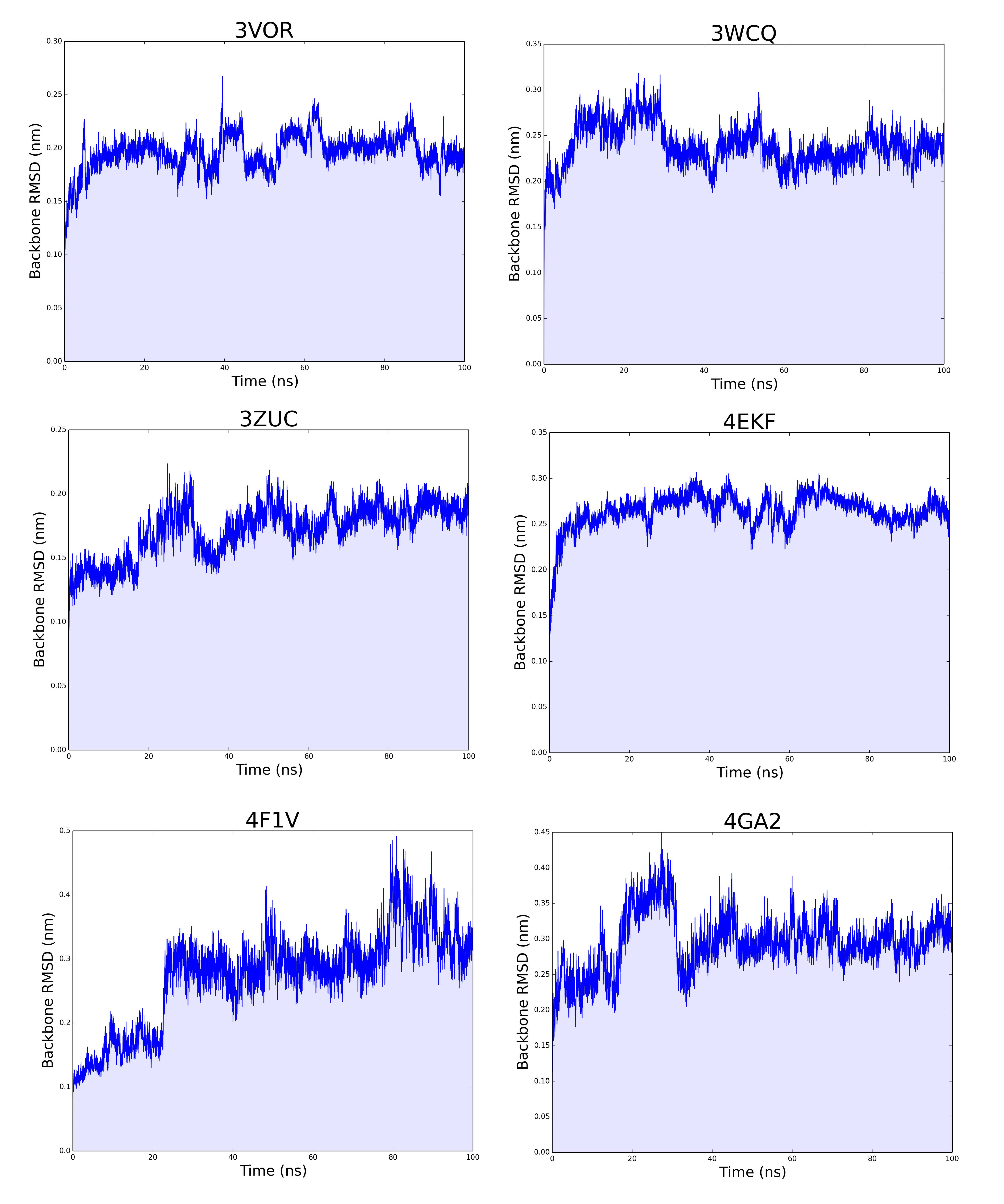

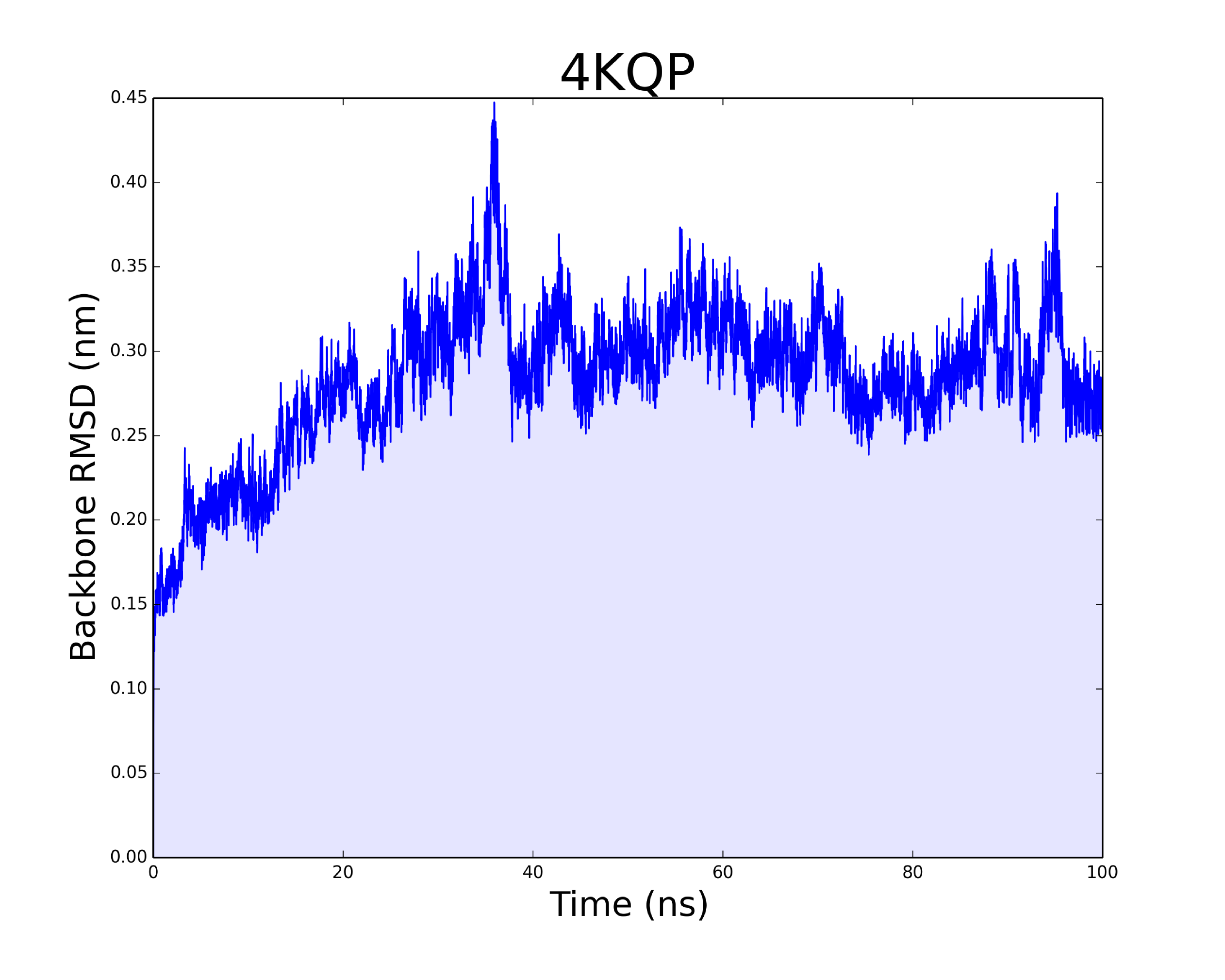
**

**Supplementary Figure 2**

β-Strand residue with 1 x NCI_C=O_, highlighting NH_bb_ () and a potential C5 hydrogen bond (dashed arrow, ) between a carbonyl group and the NH group of the same amino acid residue.


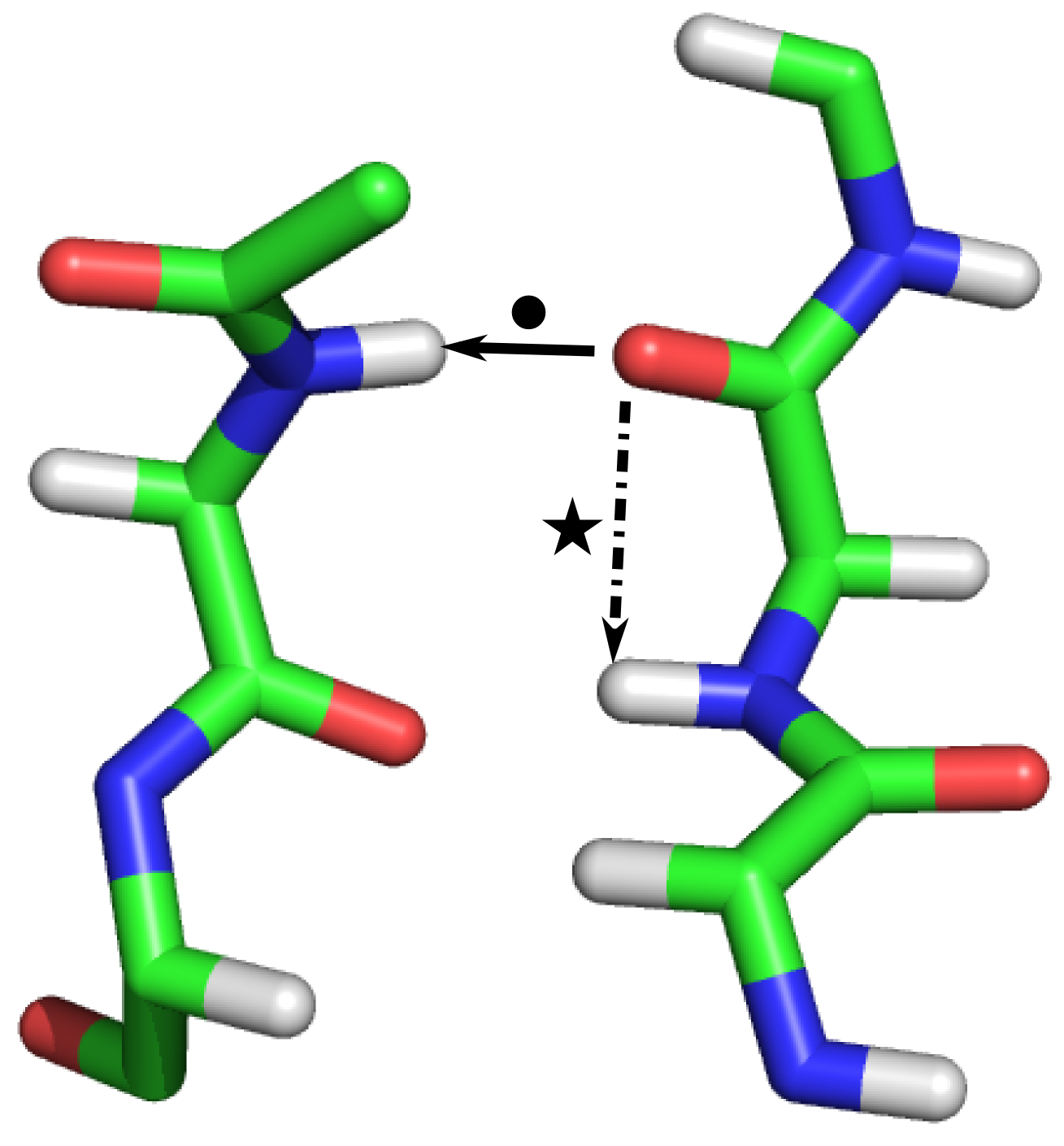


Supplementary File 1. MySQL data dump of all NCI in the database can be downloaded from <http://coiledcoils.chm.bris.ac.uk/nci/ProtSci.sql.gz>

Supplementary File 2. Summary tables of NCI types and numbers on a per-PDB basis, and PDB/residue information used to produce Figure 4 can be downloaded from <http://coiledcoils.chm.bris.ac.uk/nci/SupplementaryFile2.xlsx>
